# Impact of testicular sperm extraction and testicular sperm aspiration on gonadal function in an experimental rat model

**DOI:** 10.1590/S1677-5538.IBJU.2016.0652

**Published:** 2018

**Authors:** Leocácio Venícius Sousa Barroso, Ricardo Reges, João Batista Gadelha Cerqueira, Eduardo P. Miranda, Rafael Jorge Alves de Alcantara, Francisco Vagnaldo F. Jamacaru, Manoel Odorico de Moraes, Maria Angelina da Silva Medeiros, Lúcio Flávio Gonzaga-Silva

**Affiliations:** 1 Divisão de Urologia, Universidade Federal do Ceará, CE, Brasil; 2 Departamento de Farmacologia, Universidade Federal do Ceará, CE, Brasil

**Keywords:** Infertility, Hypogonadism, Testosterone

## Abstract

**Purpose:**

To assess the impact of sperm retrieval on the gonadal function of rats with impaired spermatogenesis by comparing testicular sperm extraction (TESE) to aspiration (TESA). The efficacy of these procedures to sperm obtainment was also compared.

**Materials and Methods:**

A pilot study showed impaired spermatogenesis, but normal testosterone (T) production after a bilateral orchidopexy applied to 26 rats, which were randomly assigned into four groups: TESE (n=7), TESA (n=7), SHAM (n=6) and Control (n=6). The T levels were measured through comparative analysis after the orchidopexy.

**Results:**

There was no statistical difference in the animal's baseline T levels after orchidopexy in comparison to the controls: the TESE and TESA groups, 6.66±4.67ng/mL; the SHAM group (orchidopexy only), 4.99±1.96ng/mL; and the Control, 4.75±1.45ng/mL, p=0.27. Accordingly, no difference was found in the postoperative T levels: TESE, 5.35±4.65ng/mL; TESA, 3.96±0.80ng/mL; SHAM, 3.70±1.27ng/mL; p=0.4. The number of sperm cells found through TESE (41.0±7.0) was significantly larger than that found through TESA (21.3±8.1, p=0.001). Moreover, higher tissue weight was found through TESE (0.09±0.02g versus 0.04±0.04g, p=0.04).

**Conclusions:**

The testicular sperm capture performed in rats through extraction or aspiration, after orchidopexy, did not significantly decrease the T levels. The amount of sperm found through testicular sperm extraction was higher than that through testicular sperm aspiration.

## INTRODUCTION

The male factor is the only etiology in approximately 30% of the conjugal infertility (CI) assessments, which turns it into a contributing factor to at least 50% of couples seeking treatment (1). Oligospermia and azoospermia are the most commonly detected seminal abnormalities (2), but azoospermia - which can be classified as obstructive or non-obstructive - accounts for 14% of all men subjected to fertility assessments (3). Before 1992, the only options available for couples whose men had non-obstructive azoospermia were ‘adoption’ or ‘insemination’ by donor semen. However, the development of assisted reproduction techniques using intracytoplasmic sperm injection (ICSI) has changed the paradigm in the treatment of infertile men. Therefore, testicular tissue exploration has become not only a diagnostic tool, but a therapeutic procedure to collect sperm to be used in ICSI (4).

Sperm retrieval approaches include open testicular biopsy, percutaneous or microsurgical epididymal extraction, and testicular sperm extraction, with or without the use of a microscope (5). However, multiple testicular procedures can result in significant testicular parenchyma loss and in blood supply impairment, which may ultimately result in testicular atrophy (6). Moreover, there is inevitable Leydig cell loss after large testicular tissue amounts are removed during such procedures. The decreased number of Leydig cells can reduce serum testosterone (T) levels and result in testosterone deficiency (TD), which may cause osteoporosis, increased insulin resistance, depression and erectile dysfunction (7). Therefore, long-term testosterone therapy (TT) may be required after testicular biopsies (8). Many studies in recent years focus on potential testicular sperm uptake, but just a few have assessed the consequences of sperm extraction on the gonadal function.

The aim of the present study was to assess the impact of sperm retrieval on the gonadal function of rats subjected to experimental orchidopexy, by comparing the testicular sperm extraction (TESE) to the testicular sperm aspiration (TESA). Comparing the efficacy of these procedures for sperm obtainment was also a goal.

## MATERIALS AND METHODS

### 

#### Animal Grouping and Experimental Orchidopexy

Male adult Wistar rats, initially weighing 320-370g, were used in the experiment. The animals were kept in 40×32×17cm cages, under light/dark cycles (12/12h), and had free access to water and food. After estimating a difference between means in treatment and control groups of 30% and a coefficient of variation of 20% due to biological differences between animals, we defined that a minimum of 5 animals per group would be required (9). The present research was approved by the local Ethics Committee on Animal Research (protocol number 77/2012). The rats were initially subjected to orchidopexy, which is known as an experimental model for impaired spermatogenesis (10, 11). The animals were anesthetized with ketamine (80mg/kg) and xylazine (6mg/kg), before the surgical procedure. All surgical instruments were sterile. The animals were put in supine position and, subsequently, a midline incision was made on their lower abdomen; their testicles were mobilized within the abdominal cavity. A bilateral gubernaculectomy was performed and the gubernaculums were anchored to the anterior abdominal wall using 4.0 nylon. The surgical wound was closed in 2 planes, by means of uninterrupted sutures using 4.0 nylon. The total of 20 animals were randomly assigned into the following groups, fifteen days (D15) after orchidopexy: TESE (n=7), TESA (n=7), and orchidopexy only (SHAM) (n=6). The Control group was composed of non-operated animals (n=6) for baseline T measurement. The T levels were measured either through sperm retrieval or sham procedure before (D16), in D15, and 23 days after (D23) the second surgery.

#### Orchiectomy for Spermatogenesis Impairment Confirmation

A pilot study was conducted to confirm the testicular histological changes caused by orchidopexy. Four non-randomized rats were subjected to orchiectomy at different intervals after surgery: 2 animals 7 days after; and 2, 14 days after. The bidigital presentation of the scrotum was carried out after anesthesia with ketamine (80mg/kg) and xylazine (6mg/Kg); the median longitudinal incision of the scrotum was done by means of dissection and bilateral isolation of the spermatic cord to identify the testicles. Both testicles were removed and subjected to histological analysis. The animals were sacrificed through cervical translocation at the end of the procedure.

#### Histological Assessment

Testis tissues were cut and fixed in Bouin solution for 24 hours and embedded in paraffin blocks. This procedure resulted in 5-μm-thick coronal sections. The testicle sections were stained in hematoxylin-eosin, mounted in neutral medium, and incubated in 0.1% periodic acid for 5-10 min. The slides were washed in tap water and immersed in Schiff's reagent for 15 min. Subsequently, the sections were washed in tap water for 10 min, counterstained in Mayer's hematoxylin, washed in tap water and dehydrated in graded ethanol.

The images were analyzed in a computer-assisted image analyzer system consisting of a microscope coupled to a digital video camera. The analyses were performed at x400 magnification.

#### Sperm Retrieval Techniques

**Figure 1:**
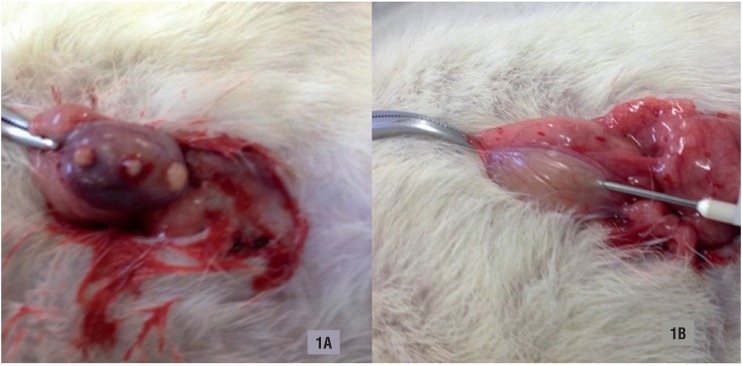
Testicular sperm retrieval procedure. A) Testicular Sperm Extraction (TESE); B) Testicular Sperm Aspiration (TESA).

1. Testicular Sperm Extraction - The animals were anesthetized in similar fashion for the second surgical procedure. After both testicles were identified, three separated 3mm incisions were performed at their upper, middle and bottom thirds using a scalpel blade 11. A light compression was made on the testicle to promote testicular tissue exposure (Figure-1A). A scissor was used in tissue collection. The samples were sent for immediate analysis. The hemostasis was performed using compression sponges. The tunica albuginea was left open. The abdominal cavity was closed with continuous suture using 4.0 nylon; 2. Testicular Sperm Aspiration - A 19-gauge needle puncture was performed in the lower pole of both testicles after testicular exposure (Figure-1B). A suction pressure using a 20mL syringe was kept to create negative pressure after the needle penetrated the tissue. The needle was pushed into different directions, 4 to 6 times. The aspirated tissue was sent to immediate analysis. The tunica albuginea at the puncture site was left open. The abdominal cavity was closed through continuous suture using 4.0 nylon; 3. SHAM Procedure - No further procedures were performed after the testicles were identified, and the abdominal cavity was closed through continuous suture using 4.0 nylon.

#### Hormonal Assessment and Sperm Processing

The blood collection was conducted after anesthesia by puncturing the retro-orbital venous sinus (12) with a capillary tube. One point five (1.5) milliliters of blood were dripped into the appropriate collector tube containing anticoagulant. The blood was subjected to centrifugation and the obtained serum was frozen and stored until the testosterone dosage was set. The collection was always performed between 8:00 and 10:00 AM. The T measurements were conducted in the Clinical Analysis Laboratory at the same institution. The plasmatic total T measurement was performed through chemiluminescence; the Beckman Coulter (Access 2 Immunoassay System/Albalab Biosystems NE/California, USA) was used in the procedure. The device was calibrated using the kit provided by the manufacturer before the analysis. All samples were assessed in duplicate and the results were expressed in nanograms per milliliter (ng/mL).

Sperm cells were collected from the testicles through TESE or TESA, after orchidopexy. Subsequently, the samples were diluted in 0.5mL of 0.9% saline solution, and kept at 37°C for analysis. Ten (10) μL of each sample were transferred to a Makler chamber for sperm cell counting through microscopy, at 100X. Two distinct samples from each testicle of each animal were separately processed, and their mean values were used for statistical analysis purposes.

### Statistical analysis

Quantitative, continuous and discrete variables were initially analyzed through the Kolmogorov-Smirnov test to verify distribution normality. Means and standard deviations were calculated for descriptive analysis, whereas parametric methods were used for analytical statistics. Comparisons between two distinct groups were performed by applying the t test in unpaired data. The t test was used in paired data to compare two different moments within the same group. Comparisons among three or more groups were conducted by means of analysis of variance (ANOVA one-way) to rank the means. The significance level 0.05 (5%) was set for all analyses. GraphPad Prism^®^ software version 5.00 for Windows (GraphPad Software, San Diego, California, USA, 2007) was used in the statistical calculations.

## RESULTS

### 

#### Histological Analysis applied to Orchiectomy Specimens

**Figure 2:**
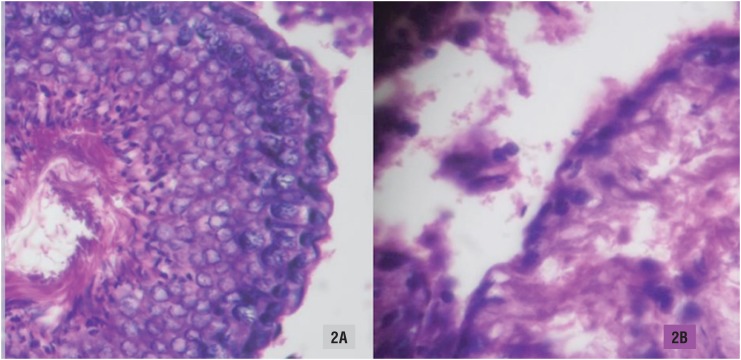
Histological analysis of an orchiectomy specimen 15 days after orchidopexy in an experimental rat model. A) Normal histology; B) Impaired spermatogenesis.

The histological examination performed in the testicles of rats subjected to orchiectomy during a pilot procedure was used to verify testicular damages after orchidopexy. No significant histological changes were observed 7 days after orchidopexy. However, a pattern of hypospermatogenesis was encountered after 15 days. The seminiferous tubule was disrupted and there was a considerable decrease in the spermatogenic cell series, as demonstrated in Figure-2. Therefore, we concluded that spermatogenesis was significantly impaired 15 days after orchidopexy.

#### Sperm retrieval impact on the T levels

Postoperative serum T levels (D23) in rats belonging to the TESE group (5.35±4.65ng/mL) were similar to the control rats (4.75±1.45ng/mL, p=0.8) and to the SHAM group, 15 days after orchidopexy (4.99±1.96ng/mL, p=0.9). Likewise, there was no significant difference between the postoperative serum T levels (D23) in rats from the TESA Group (3.96±0.80ng/mL) and the controls (4.75±1.45ng/mL, p=0.2). The comparison between postoperative T levels in rats belonging to the TESA (3.96±0.80ng/mL) and SHAM groups (4.99±1.96ng/mL) on (D15) showed no significant difference (p=0.3). The postoperative T levels in the SHAM groups (3.70±1.27ng/mL) did not significantly differ from the controls (p=0.2). Comparisons among TESE, TESA and SHAM were performed and no significant difference was found (p=0.6). All T measurements performed in different time points are displayed in Table-1.

**Table 1:** Testosterone levels among experimental animal groups.

T Levels	Preoperative	Postoperative	p
TESE	6.66±4.67*	5.35±4.65	0.8
TESA	6.66±4.67*	3.96±0.80	0.2
SHAM	4.99±1.96	3.70±1.27	0.6
Control	4.75±1.45	-	-

* Preoperative T levels of groups TESE and TESA were analyzed together when compared to controls.

**TESE =** Testicular Sperm Extraction; **TESA =** Testicular Sperm Aspiration; **SHAM =** Rats submitted to orchidopexy only + sham procedure.

#### Sperm retrieval outcomes

The absolute number of sperm cells found through TESE (41.00±7.01) was significantly higher than that found through TESA (21.33±8.14) (p=0.001). In addition, the testicular tissue weight found through TESE (0.09±0.02) g was significantly higher than that found through aspiration (0.09±0.02g versus 0.04±0.04g, p=0.04).

## DISCUSSION

The sperm retrieval procedures performed in the present study did not significantly decrease the T levels in the experimental rat models presenting impaired spermatogenesis after orchidopexy. This procedure preserved T production since no significant difference was observed between the T levels measured in rats subjected to orchidopexy and in non-operated animals, despite its negative effects on sperm production. Furthermore, TESE was better than TESA in retrieving sperm cells and testicular tissue.

One of the biggest concerns about performing surgical sperm retrieval in humans lies on the long-term impact on T production. Controversial results have been published, but there is consensus about the fact that the micro-TESE may impair Leydig cell functioning (13–15). Accordingly, TESA has emerged as an alternative to alleviate some possible negative effects, as well as a viable and effective ART option. The present results contradict the aforementioned concept and presents TESE as the most effective procedure in terms of hormonal function damage.

The present experimental protocol adds value to the literature, because it aims at representing a subfertile population. There is no rationale to investigate these procedures in rats presenting normal spermatogenesis if one considers that only men, with significant spermatogenesis alteration, are subjected to testicular sperm extraction. Different experimental models promoting spermatogenesis impairment have been previously described; the most recognized models include chemotherapy infusion (16), drug-induced cryptorchidism (17) or surgical orchidopexy (10, 11). Orchidopexy was chosen as procedure in the current experiment because it is a simple and inexpensive method of proven efficacy. The testicular volume reduction in rats, followed by the herein performed orchidopexy, can be visually observed, although further assessment applied to these findings may extrapolate the current scope. The initial spermatogenesis changes shown in the testicular histological analysis were already observed 7 days after orchidopexy, and it became even more evident on D15. Previous studies have shown the presence of degenerative changes in spermatogenesis of rats presenting cryptorchidism (18, 19). Rossi et al. have performed histological analysis in the intra-abdominal testicles after orchidopexy and found spermatogenesis impairment, Sertoli-cell-only syndrome and maturation arrest in the 15^th^ postoperative day (20). Their findings validate the chosen time-frame applied to the present protocol.

Accurate serum T measurements remain a challenge to both research and clinical setting. The recommended serum testosterone methodology is based on high-performance liquid chromatography and on tandem mass spectrometry (21). Unfortunately, these technologies are limited to certain centers, due to their high cost and complex pre-dialysis processes. Recent studies have shown that they may lack accuracy in specific situations, notably in low concentrations, as found in women and children; thus, the chemiluminescence was used in the present analysis. However, several steps, such as previous apparatus calibration, duplicate dosages and blood collection in normal rats, were taken to avoid methodological errors.

Kerr et al. subjected adult rats to unilateral orchidopexy for one, two and four weeks (10). The morphological changes in the Leydig cells in the abdominal and scrotal testicles were measured using the morphometric approach applied to the volume and number of Leydig cells per testicle. Although a volume reduction of approximately 30% was found in the intra-abdominal testicles two weeks after surgery, the number and volume of Leydig cells and serum T levels were similar. Similarly, there was no significant difference between preoperative serum T concentrations in rats belonging to the TESE and TESA (after orchidopexy) groups in comparison to the controls. The T level changes after sperm retrieval could not be attributed to orchidopexy, after the experiment was carried out.

Other authors have studied the serum T changes in animals subjected to testicular biopsy (17) and found T concentration reduction in rats who underwent TESE. However, such decrease was clearly related to the amount of extracted tissue. The current study did not find significant difference between the T levels in rats subjected to orchidopexy and TESE, to orchidopexy alone, and the control rats. Therefore, it is possible stating that the TESE experiment did not lead to decreased T levels. This contradictory finding may result from the small volume of extracted tissue through TESE (0.09g of extracted tissue, on average), although such amount was significantly higher than that extracted through TESA. Likewise, TESA did not cause T level changes in the rats after the orchidopexy in the present study. No significant difference was observed between the T concentration measured in rats subjected to orchidopexy and TESA, and that found in non-operated animals, or in animals subjected to orchidopexy, alone.

Authors reported that the postoperative T levels in rats, after orchiectomy, remain at 26.4 to 81.5% of their baseline level. Interestingly, there are reports stating that the T levels in rats, after orchiectomy, fail to become undetectable or to reach very low levels (22). A pioneer study found that increased T and androstenedione levels are maintained by the production of adrenal glands after the rats are castrated, but the same result is not observed in humans (23). These studies may explain why no significant T level reduction was found after the TESE and TESA procedures were herein applied. Moreover, the hypothesis of having the surgical manipulation as the responsible for T level changes between groups was rejected, since the T levels in the SHAM group did not differ from the control. Multiple open biopsies (three in each testicle) were chosen and the amount of sperm obtained through TESE was larger than that through TESA in the present study. Despite the larger amount of tissue removed through TESE, no significant differences were observed in the T levels.

The small number of animals in each group turns out to be a limitation to the present study. For ethical reasons, sampling was limited to the minimum number required for reliable statistical analysis. The adoption of an automatic method to assess serum T levels through chemiluminescence was another limitation to the study, because it could have overestimated the values.

There are multiple sperm-capturing approaches. The testicular microdissection technique for sperm extraction developed by Schlegel allowed improving the sperm uptake rates and reducing the testicular damage in comparison to the standard open testicular biopsy (24). Despite the technical improvement, spermatogenesis foci were not found in all the studied cases. Locating these sperm-producing islands may require considerable time (8). Future studies are required to develop techniques to improve the identification of the limited foci, to enhance successful capture rates, as well as to reduce the surgery time and the extracted-tissue volume rates. In addition, the experimental models for microdissection TESE in rats are still required, as the manipulation of their <0.5mm tunica albuginea remains a challenge.

## CONCLUSIONS

The testicular sperm capture in rats, after orchidopexy, either through extraction or aspiration, did not significantly decrease the T levels. The amount of sperm found through testicular sperm extraction was higher than that through testicular sperm aspiration.
